# Fibrosarcomatous changes and expression of CD34+ and apolipoprotein-D in dermatofibrosarcoma protuberans

**DOI:** 10.1186/2045-3329-2-4

**Published:** 2012-01-27

**Authors:** Emanuela Palmerini, Marco Gambarotti, Eric L Staals, Licciana Zanella, Gabriela Sieberova, Alessandra Longhi, Marilena Cesari, Stefano Bonarelli, Piero Picci, Pietro Ruggieri, Marco Alberghini, Stefano Ferrari

**Affiliations:** 1 Chemotherapy, Musculoskeletal Oncology Department, Istituto Ortopedico Rizzoli, via Pupilli 1, Bologna, 40136, Italy; 2 Surgical Pathology, Musculoskeletal Oncology Department, Istituto Ortopedico Rizzoli, via Pupilli 1, Bologna, 40136, Italy; 3 Orthopaedic Surgery, Musculoskeletal Oncology Department, Istituto Ortopedico Rizzoli, via Pupilli 1, Bologna, 40136, Italy; 4 Laboratory of Oncologic Research, Musculoskeletal Oncology Department, Istituto Ortopedico Rizzoli, via Pupilli 1, Bologna, 40136, Italy

**Keywords:** dermatofibrosarcoma protuberans, soft tissue sarcoma, Apolipoprotein-D, CD34

## Abstract

**Background:**

Dermatofibrosarcoma protuberans (DFSP) is a relatively common soft-tissue tumor. A more aggressive appearing fibrosarcoma may arise in DFSP, changing its biological behavior. CD34 and apolipoprotein-D are highly expressed in DFSP, but their prognostic significance is uncertain.

**Methods:**

DFSP and fibrosarcomatous-DFSP (FS-DFSP) patients referred to our institute between 1982 and 2009 were identified. Fibrosarcomatous changes, expression of CD34 and apolipoprotein-D were evaluated.

**Results:**

40 patients, (median age 43 years, 55% males) were identified. Tumor was located in the limbs in 60%, in the trunk in 40%. Thirty-seven patients had localized and 3 had metastatic disease. Thirteen (32%) patients were FS-DFSP. All but one underwent surgery with adequate surgical margins in 72%. 7 FS-DFSP received also radiotherapy (RT). Chemotherapy was administered to 3 patients with FS-DFSP. With a median follow-up of 49 months, the 5-OS was 90%. Local recurrence rate was 23%: 42% FS-DFSP, 15% DFSP. Metastases developed in three FS-DFSP patients. The 5-year EFS was 70% in localized patients. Histology (DFSP 75% vs. FS-DFSP 52%, p = 0.002), surgical margins (adequate 74% vs. inadequate 55%, p = 0.02), site (limb 47% vs. trunk 100%), CD34 expression (CD34 positive: 70% vs. CD34 negative: 33%, p = 0.05), and apolipoprotein-D expression (Apo-D positive: 73% vs. Apo-D negative: 33%, p = 0.02) influenced the 5-year EFS, whereas sex, use of RT or number of previous surgical treatments did not.

**Conclusions:**

Patients with DFSP have a high survival probability. Site, adequate surgical margins, presence of the fibrosarcomatous component, lack of CD34 expression and apolipoprotein-D influence outcome.

## Background

Dermatofibrosarcoma protuberans (DFSP) is a low grade malignant mesenchymal tumor that typically arises in the dermis of the trunk and proximal extremities [1]. DFSP represents 1 to 6% of all soft tissue sarcomas (STS) [2,3] and its frequency of detection slowly has increased over time [4]. DFSP is characterized by latency of initial diagnosis, slow infiltrative growth and a high rate of local recurrence if not adequately treated. Death due to metastatic disease is very rare (< 5%) [5]. Histologically, DFSP is usually characterized by uniform spindle shaped cells with elongated neuroid nuclei, proliferating in a storiform growth pattern, infiltrating subcutaneous tissues with a "honeycomb" appearance. In rare cases DFSP shows areas with high-grade fibrosarcomatous changes (more than 5 mitoses/10 HPF, a fascicular growth pattern, increased cellularity and atypia). When dedifferentiated areas represent more than 5% of tumor tissue, the lesion is classified as fibrosarcomatous ("high-grade") dermatofibrosarcoma protuberans (FS-DFSP) [1]. The prognostic influence of the fibrosarcomatous component of FS-DFSP has been debated [1].

Immunohistochemically, most DFSP stain positively for CD34, whereas, FS-DFSP are CD34 positive in about half of cases [6]. Apolipoprotein-D (Apo-D), a glycoprotein component of human plasma lipid transport system, has been found to be highly expressed in DFSP by gene arrays [7] and it has been proposed as a novel marker for this neoplasm [8]. The role of Apo-D in DFSP is unknown.

Most DFSPs are cured by wide surgical excision [1]. In some experienced centers, Mohs micrographic surgery (MMS) is routinely used instead of wide excision [5]. Radiotherapy also has been employed [9]. Chemotherapy has been adopted in FS-DFSP patients. Distant metastases generally occur as late sequela following repeated local recurrences. In some of these patients, imatinib has been used successfully [10,11]. The target of imatinib in these tumors is the product of a chromosomal translocation that involves chromosomes 17 and 22, resulting in fusion of the collagen type I αI (*COL1A1*) and platelet-derived growth factor ß (*PDGFß*) gene, in both DFSP and FS-DFSP [12]. A FISH probe for *COL1A1/PDGFB *detection is not yet commercially available.

The objective of this study was to retrospectively examine all DFSP and FS-DFSP patients treated at our institution in order to identify tumor- and treatment-related factors influencing survival.

## Methods

After obtaining Institutional Review Board approval, all patients diagnosed with DFSP and FS-DFSP between 1982-2009 were identified from the database of the Pathology Department of Musculoskeletal Oncology at the Rizzoli Institute. Two pathologists (M.G. and G.S.) reviewed the slides. Tumors were classified according to the Enzinger & Weiss criteria: FS-DFSP were identified by the presence of fibrosarcomatous changes (more than 5 mitoses/10 HPF, a "fascicular" growth pattern, increased cellularity and atypia) in at least more than 5% of tumor tissue [1]. Immunohistochemical expression of CD34 (Qbend-10, 1-100 dilution, Dako, Carpinteria CA, USA) and Apo-D (36C6, 1-200 dilution, Novocastra, New-castle-on-Tyne, UK) were assessed by two pathologists (M-G. and L.Z.) in all patients with adequate tumor tissue. Detection of the two antibodies was performed on a Dako automated immunostainer with universal detection kit streptavidin biotin-alkaline phosphatase/red/detection system Dako after heat (Apo-D) and enzyme (CD34) induced antigen retrieval.

All patients surgically treated at our institution with histological diagnosis confirmed were included in the analysis. After 1986, the staging consisted of a computed tomography scan (CT-scan) and/or magnetic resonance imaging of the primary lesion, and a chest CT-scan; other specific tests (bone scan, abdomen CT-scan) were performed only in the case of clinical suspicion. Prior to 1986, a plain chest X-ray and ultrasound of the lesion were performed. Assessment of the surgical margins was based on both the pathology report and the description of the surgical excision. All patients were followed-up with ultrasound, computer tomography or magnetic resonance imaging studies at three to four-months intervals for at least two years, and subsequently at six-months intervals for another three years.

Pattern of recurrence for localized patients were defined as follow: local recurrence, when tumor relapse was confined to the primary tumor area; metastases, for distant only metastases: local recurrence plus metastases for local and distant recurrence.

### Statistical Analysis

The following parameters were examined for prognostic value in patients with localized disease: patient sex, tumor anatomic site, surgical margins, histology, CD34 and Apo-D expression, number of previous surgical treatment, use of radiotherapy.

The following categories were compared: tumor site (extremity: at or distal to the shoulder joint and in the groin or leg; trunk: proximal to the shoulder joint and the groin); surgical margins (adequate: wide or radical; inadequate: intralesional, marginal or contaminated margins, according to Enneking's classification)[13]; histology (DFSP vs. FS-DFSP); CD34 and Apo-D expression (positive or negative), number of previous operations (0 or ≥ 1); adjuvant treatments (radiotherapy performed within 3 months after tumor excision).

We analyzed overall survival (OS) and event-free survival (EFS). OS time was calculated from the time of admission at our Institute to death or last follow-up visit. EFS time was calculated from the time of admission at our Institute and the occurrence of an event. An event was defined as local recurrence, distant recurrence or death (disease-related or unrelated). All time-to-event end points were modeled using the method of Kaplan and Meier and analyzed by the log-rank test. The results of the Cox model analysis are reported as relative risks (RRs) and 95% confidence intervals (CIs).

## Results

**Figure 1:**
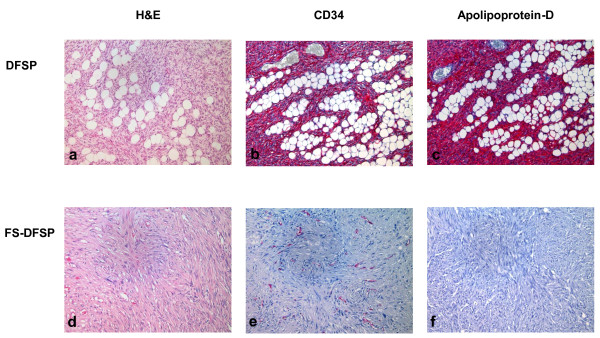
**Histology**. Bland appearing spindle cells arranged in monotonous storiform pattern, infiltrating between lobules of fat in an honeycomb pattern in DFSP (H&E; panel a). CD34 (panel b) and Apolipoprotein-D (panel c) expression in DFSP. Malignat fibrous histicitoma-like areas in DFSP (DFSP-FS) (panel d). Loss of expression of CD 34 (panel e) and Apolopoprotein-D (panel f).

A total of 51 consecutive patients with histologic diagnosis of DFSP or FS-DFSP made between 1982 and 2009 were identified. Eleven patient were consultation cases, therefore 40 patients were included in the study (Table 1). Reason for admission at our institution was: a new diagnosis in 4 patients (10%), local recurrence in 19 (47.5%), scar re-excision 14 (35%) and distant (lymph node) metastases plus local recurrence in 3 patients (7.5%). In 13 (32.5%) patients, sarcomatous changes were documented. In 26 of the 40 evaluable patients tissue samples were available for immunohistochemical assessment of the expression of CD34+ and Apo-D (Figure 1, Table 2).

**Table 1:** Distribution of Clinical Pathologic, and Treatment Characteristics

Number of patients identified	40
Sex	
Female	18 (45%)
Male	22 (55%)
Site of primary	
Limbs	24 (60%)
Trunk	16 (40%)
Age	
Median (min-max)	39 yrs (4-80)
Histology	
DFSP	27 (67.5%)
FS-DFSP	13 (32.5%)
Metastases at presentation	
None	37 (92.5%)
Limphonodal	3 (7.5%)
Previous surgical treatment	
Yes	36 (90%)
No	4 (10%)
Median n° of operations (min-max)	1 (1-5)
Margins *	
Wide	28 (72%)
Marginal	5 (13%)
Intralesional	6 (15%)
Radiotherapy	
Yes	11 (27%)
No	29 (73%)
Chemotherapy	
Yes	3 (7.5%)
No	37 (92.5%)

* margins were available in the 39 patients surgically treated

**Table 2:** Immunoistochemical Expression of CD34 and Apolipoprotein D*

	All (26 pts)	DFSP (15 pts)	FS-DFSP (11 pts)	
	n (%)	n (%)	n (%)	
CD34				
Yes	20 (77)	13 (87)	7 (63)	p = 0.3
No	6 (23)	2 (13)	4 (37)	
Apolipoprotein-D				
Yes	17 (65)	11 (73)	6 (55)	p = 0.4
No	9 (35)	4 (27)	5 (45)	

* in 26 patients; pts: patients; DFSP: dermatofibrosaroma protuberans; FS-DFSP fibrosarcomatous-DFSP

### Local Treatment

Most patients (36/40) had already been surgically treated. Thirty-nine patients (97.5%) underwent surgery at our Institute. The amputation rate was 8% (3/39). One patient refused the planned surgical treatment (amputation) and was lost to follow-up. Adequate surgical margins were detected in 28 of 39 (72%) surgically treated patients (Table 1). Radiotherapy was administered to 11 patients ***(7 with FS-DFSP).

### Chemotherapy

Adjuvant ifosfamide and epirubicin was administered to 3 patients, all had FS-DFSP. In two cases it was employed after excision of a local recurrence and in one case following an amputation for local recurrence and nodal metastases.

### Outcome

**Figure 2:**
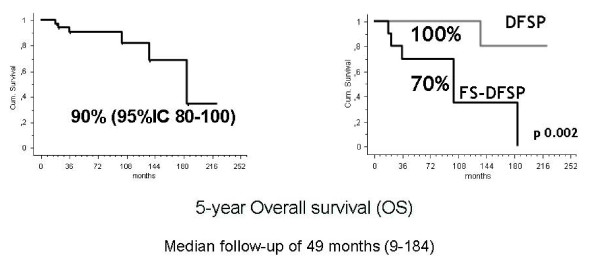
**Overall Survival.** Five-year overall survival (OS) in 40 DFSP patients with localized disease and metastatic disease at presentation (left). Five-year overall survival (OS) according to histology (right).

**Figure 3:**
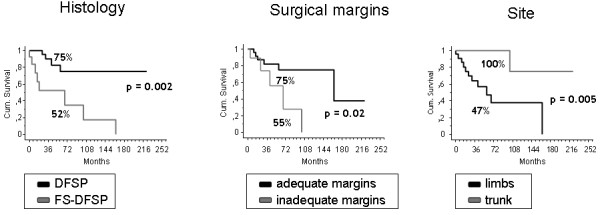
**Five-year event free survival (EFS) according to histology, adequacy of surgical margins and site**.

Two patients were lost to follow-up. With a median follow-up of 49 months (9-184), the 5-year overall survival (OS) was 90% (95%CI 80-100) (Figure 2). The 5-year event-free survival (EFS) was 69% (95%CI 51-87) in localized patients. Histology (DFSP 75% (95%CI 53-97) vs. FS-DFSP 52%,(95%CI 21-83), p = 0.002), surgical margins (adequate 75% (95%CI 55-95) vs. inadequate 55% (95%CI 16-95), p = 0.02), site (extremity 47% (95%CI 22-73) vs. trunk 100%, p = 0.005), influenced the 5-year EFS, whereas sex, use of RT or number of previous surgical treatments did not (Figure 3). Multivariate analysis (via Cox regression) was performed. Site (extremity RR 36.9, 95%CI 2.1 to 621.7, p = 0.01), sex (female RR 0.08, 95%CI 0.009 to 0.7, p = 0.02) and histology (FS-DFSP RR 0.1, 95%CI 0.01 to 0.8, p = 0.03) independently influenced EFS.

### CD34 and apolipoprotein-D expression

**Figure 4:**
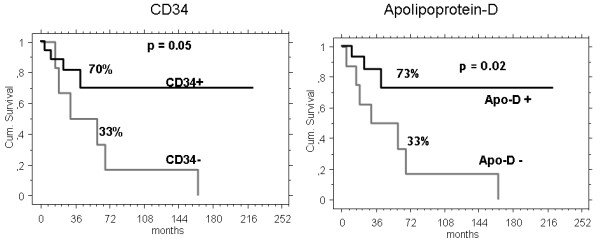
**Five-year event free survival (EFS) according to CD34 and Apolipoprotein-D expression**.

CD34 and Apo-D expression in DFSP and FS-DFSP is shown in Table 2. The 5-year EFS was 70% (95%CI 43-96) in CD34 positive vs. 33% (95%CI 0-71) in CD34 negative patients, (p = 0.05), and 73% (95%CI 45-100) in Apo-D positive vs. 33% (95%CI 0-69) in Apo-D negative patients, (p = 0.02) (Figure 4). We did not find significant difference according to histology, site or margins between the two groups. When CD34 and Apo-D were included in the multivariate analysis they lost their prognostic significance.

### Pattern of failure in patients with localized disease

Incidence of local recurrence was 23% (9/39) overall: 42% in FS-DFSP and 15% in DFSP. All relapses occurred in tumors of the limbs, while no recurrences were observed in tumors of the trunk. Three patients with local recurrence developed also pulmonary metastases; all of them presented with FS-DFSP histology; none of the patients with DFSP developed metastases. Local recurrence occurred in 5/28 (18%) patients with adequate margins and in 4/11 (36%) patients with inadequate (intralesional or marginal) margins. None of patients with inadequate margins developed distant metastases.

## Discussion

DFSP is a soft tissue malignancy that often shows extensive local invasion but rarely metastasizes [14]. DFSP can lead to significant morbidity from repeated surgical excisions, but infrequently leads to death [3,5,6,15,16].

Dermatofibrosarcoma protuberans with fibrosarcomatous dedifferentiation (FS-DFSP) represents an more aggressive appearing variant of DFSP, in which the prognostic influence of the fibrosarcomatous component is still debated. In our series the high grade variant represented almost a third of the cases, most likely due to a referral center selection bias.

In this study the FS-DFSP patients developed distant recurrence in 23% and local recurrence in 42%, while DFSP was associated with a 15% of local recurrence rate and no distant metastases. In other series the rates of local and distant recurrence are similar (Table 3) [6,15-18].

**Table 3:** Local and distant recurrence rate in FS-DFSP

**Ref**.	Pts n°	Distant	Local
Palmerini et al., *current study*	13	23%	42%
Mentzel T et al., *AJSP 1998 *[*6*]	41	15%	58%
Bowne WB et al., *Cancer 2000 *[*15*]	25	8%	52%
Fiore M et al, *JCO 2005 *[*16*]	7	28%	28%
Abbott JJ et al. *AJSP 2006 *[*17*]	41	10%	20%
Goldblum JR et al., *AJSP 2000 *[*18*]	18	0%	22%

Furthermore the presence of high grade fibrosarcomatous changes in our series is statistically associated with inferior EFS. Therefore, fibrosarcomatous changes in DFSP represent a clinically relevant form of tumor progression. Several authors suggested that fibrosarcomatous areas within DFSP are associated with higher local recurrence and distant metastases rate [6,18-21]; however, in these studies, the status of surgical margins was not clear. Goldblum studied 18 FS-DFSP patients with adequate margins and reported a relatively low local recurrence rate (22%) (Table 3) and no distant recurrence. [19]. In our series, 3 out of 13 patients with FS-DFSP and wide margins developed distant relapse. We believe that an higher rate of local and distant recurrence in FS-DFSP compared to DFSP could be related to the presence of high-grade fibrosarcomatous changes and not only to the status of margins. The different results from our and Goldblum studies may be due to differences in patient selection.

Apo-D and CD34 were recently employed as useful markers in differentiating superficial tumors, including DFSP [22]. In our series, the positivity to both membrane markers was documented in about 2/3 of patients. The expression is lower in FS-DFSP compared with DFSP. EFS at 5 years was significantly better in CD34 positive patients. Very similarly, Apo-D positive patients, had an EFS at 5 years of 73%, compared to 33% in Apo-D negative cases. We did not find significant differences according to histology (DFSP vs. FS-DFSP), site or margins in the 2 groups. The multivariate analysis did not confirm the independent prognostic significance of the expression of Apo-D and CD34, possibly because of the small sample size. To our knowledge, our series is the first addressing their prognostic role in the subset of DFSP or FS-DFSP. Our observation, an increased risk of local recurrence in patients with lower expression of these markers, should be considered in the planning of the surgical strategy.

## Conclusions

Patients with DFSP have a high survival probability. In this series, limb location, inadequate surgical margins and fibrosarcomatous changes were associated with higher local and metastatic recurrence. The role of adjuvant therapies has yet to be defined. Loss of CD34+ and/or Apolipoprotein D expression are associated with a worse prognosis in this series.

## Competing interests

The authors declare that they have no competing interests.

## Authors' contributions

The work presented here was carried out in collaboration between all authors. PE, GM and SLE defined the research theme; LZ, GM, SG and MA: carried out the pathological studies and Immunohistochemical assays; LA, CM and SB co-worked on associated data collection and their interpretation; EP, PP, PR, AM, and FS discussed analyses, interpretation, and drafted the manuscript. All authors read and approved the final manuscript.
